# Current Concepts and Future Trends in Increasing the Benefits of Cochlear Implantation: A Narrative Review

**DOI:** 10.3390/medicina58060747

**Published:** 2022-05-31

**Authors:** Cristina Maria Blebea, Laszlo Peter Ujvary, Violeta Necula, Maximilian George Dindelegan, Maria Perde-Schrepler, Mirela Cristina Stamate, Marcel Cosgarea, Alma Aurelia Maniu

**Affiliations:** 1 Department of Otorhinolaryngology, “Iuliu Hatieganu” University of Medicine and Pharmacy, 400347 Cluj Napoca, Romania; cristina_blebea@yahoo.com (C.M.B.); neculav@yahoo.com (V.N.); maximilian.dindelegan@gmail.com (M.G.D.); mctmedic@yahoo.com (M.C.S.); rcosgarea@yahoo.com (M.C.); almacjro@yahoo.com (A.A.M.); 2 County Clinical Emergency Hospital Cluj, 400347 Cluj Napoca, Romania; 3 Institute of Oncology “Prof.Dr. Ion Chiricuta”, 400015 Cluj Napoca, Romania; pmariaida@yahoo.com

**Keywords:** cochlear implant, hearing loss, deafness, review, nanomaterials, dexamethasone

## Abstract

Hearing loss is the most common neurosensory disorder, and with the constant increase in etiological factors, combined with early detection protocols, numbers will continue to rise. Cochlear implantation has become the gold standard for patients with severe hearing loss, and interest has shifted from implantation principles to the preservation of residual hearing following the procedure itself. As the audiological criteria for cochlear implant eligibility have expanded to include patients with good residual hearing, more attention is focused on complementary development of otoprotective agents, electrode design, and surgical approaches. The focus of this review is current aspects of preserving residual hearing through a summary of recent trends regarding surgical and pharmacological fundamentals. Subsequently, the assessment of new pharmacological options, novel bioactive molecules (neurotrophins, growth factors, etc.), nanoparticles, stem cells, and gene therapy are discussed.

## 1. Introduction

In recent decades, hearing loss has become an increasingly concerning health issue. According to the World Health Organization, around 466 million people worldwide have disabling hearing loss (https://www.who.int/news-room/fact-sheets/detail/deafness-and-hearing-loss (accessed on 28 May 2022)). Hearing loss is the most common neurosensory disorder when considering all sensory deficits and carries a tremendous economic and social burden, considerably impacting patients’ quality of life.

For patients with severe hearing loss or deafness, cochlear implantation (CI) has become the gold standard of treatment and is constantly being improved and adapted to patients’ actual needs. Given the results obtained since the initial implementation in 1971 [1], cochlear implants are recognized among the best neurobionic prostheses. As CI indications extend to patients with good residual hearing, preserving this function by avoiding cochlear trauma during implantation has become a priority. After the development of the cochlear implant, many attempts were made to improve the outcomes of the procedure itself. The surgical approach of cochlear implantation, as well as the materials used and technical design, was studied to prevent electrode insertion trauma and preserve residual hearing. Clinicians and researchers also focused on other methods to reduce the inflammatory response of the inner ear following cochlear implantation (glucocorticoids, inhibitors of cell death pathways, and hypothermia). Novel biomaterials and nanomaterials are also being investigated in order to achieve a sustained and controlled drug delivery into the inner ear.

As we witness a steady increase in knowledge regarding the inner ear’s physiology and with the development of targeted therapies, many lines of research regarding CI and ways to increase its functionality are being explored. However, few of these results will actually reach mass adoption and clinical usage. Many of the novel treatment options are in accordance with the Gartner Hype Cycle for Emerging Technologies, but only a few will prove their actual benefit. Thus, we believe that a review of the current knowledge is necessary to sum up the principles that are being considered for future implementation. Through this review, we outline the surgical principles of CI, with emphasis on recent technical improvements, and discuss emerging therapies focused on improving the outcomes of cochlear implantation.

## 2. Surgical Techniques

As selection criteria for CI are continuously evolving and more patients are eligible for implantation, the preservation of residual hearing is becoming increasingly studied. Sustained trauma to the cochlea during the advancement of the electrode array was identified as a critical factor that can deteriorate residual hearing; therefore, in recent years, increasing attention has been focused on the surgical procedure.

### 2.1. Soft Surgery

The aim of preserving residual hearing led to an emphasis on avoiding mechanical trauma to the cochlea, leading to the use of the term ‘‘soft’’ surgical technique. After the first mention of the soft-surgery concept by Lehnhardt in 1993 [2], several researchers have attempted to systemize the basic principles of this concept. The intention of soft surgery is to reduce the mechanical stress transmitted to the cochlea by using drills judiciously and limiting the trauma caused by electrode advancement, as well as by limiting the accidental introduction of autologous materials (blood, bone dust, etc.) in the inner ear that could cause an intracochlear reaction [3].

Cochlear implantation most commonly begins with cortical mastoidectomy followed by posterior tympanotomy and preparation of the device well. Non-mastoidectomy approaches, such as the pericanal approach, the suprameatal approach, and the transcanal approach, still require drilling to create the groove that shelters the electrode [4]. One principle of soft surgery principles is to avoid bone dust entering the cochlea. Bone dust can trigger an inflammatory response within the scala media and alter residual hearing.

Noise and vibration during surgical drilling has become a concern for the potential reduction in residual hearing. Pau et al. determined that drilling at the level of the promontory exceeds 100 dB SPL, with further increased SPL when the endosteal layer is exposed, and exceeding 130 dB SPL when the round window membrane is touched by the drill [5]. Vibration generated by the burr during mastoidectomy or inadvertent contact with the ossicular chain is considered to induce an auditory threshold shift, possibly due to cellular and stria vascularis damage [6] Blood, similarly to bone dust, is regarded as a foreign body; hence, avoiding its entry into the vestibule is critical in preventing further sensorineural hearing loss. Although one of the core soft-surgery principles is to avoid contamination of the vestibule, there is little evidence to suggest that cochlear blood contamination can cause neurosensorial hearing loss. Avoiding perilymph suctioning or leakage is a primary concern during surgery, as it can contribute to possible cochlear damage due to changes in the endocochlear potential [7] or even lead to mechanical damage to the basilar membrane [8].

The use of hyaluronic acid (HA) in cochlear implantation is not a new concept, as Donnelly demonstrated it in 1995 by using HA to facilitate electrode insertion into fresh human temporal bone specimens [9]. In the same year, Roland et al. assessed the toxicity of HA in vivo by injecting it into the ears of guinea pigs. The authors found that HA did not affect the spiral ganglion neurons, which had even preserved the dendrite and axon histology [10]. Other articles also noted that HA lacks any toxic effects on the inner ear [3,11]. Given the safety profile, many studies have used HA to reduce friction forces during electrode insertion and to keep a seal on the perilymph inside the cochlea following electrode insertion [11,12,13,14]. Although HA reduces friction and seals off the perilymph, some studies revealed no significant benefit with respect to hearing preservation [15]. Other lubricants mentioned in the literature are oxycellulose (hydroxypropyl methylcellulose) and glycerin. The former is not metabolized, leading to a foreign body reaction inside the cochlea, whereas the latter has a high impedance, which interferes with the electrode’s electric stimulation properties. Both arguments are strong enough that each substance loses its clinical utility [10].

Another emerging surgical concept is that of partial electrode insertion in patients with residual hearing who are candidates for electric–acoustic stimulation. Lenarz et al. hypothesize that partial electrode insertion could offer protection for the lower frequencies, and if hearing loss progresses postoperatively, the electrode can be further inserted [16].

Intraoperative electrocochleography (ECochG) has been utilized during CI surgery and can provide the surgeon objective feedback during electrode insertion. Although it is not a method used to reduce inflammatory reaction by itself, whenever ECochG amplitude or phase responses are modified, the surgeon can adjust the course of the electrode in order to obtain normal intracochlear potentials and limit potential physical trauma to the cochlea [17].

In addition to the soft surgery principles, it has been hypothesized that applying hypothermia to the surgical site has the potential to reduce immediate and delay secondary hearing loss. In preclinical studies, local application of controlled hypothermia rendered a protective effect by decreasing the auditory function loss associated with electrode insertion [18,19]. Taking into consideration existing knowledge regarding hypothermia, ongoing studies on human temporal bones consider cooling probes and iced irrigation, with temperature distribution measurements at the level of the round window and different cochlear levels [20,21,22].

### 2.2. Cochleostomy vs. Round Window Insertion

The cochlear implant electrode array is positioned in the scala tympani when normal anatomy is present. The round window membrane or a basal turn cochleostomy are the two most common ways to access the scala tympani in order to insert the electrode array [3]. Each has its own set of benefits and limitations.

The round window membrane (RWM) is a natural boundary of the scala tympani. It has become an increasingly popular route for electrode insertion in cochlear implantation, particularly for hearing preservation. Preliminary comparisons revealed that the round window approach causes much less traumatic than insertion through a cochleostomy [23]. Drilling is usually not required to reach the scala tympani; therefore, noise exposure and the risk of bone dust entering the cochlea are limited [24]. Even when drilling is involved in eliminating the bony overhang of the round window niche, it takes less time and surface exposure than performing a cochleostomy. A cochleostomy requires drilling of an overlying segment of the promontory. The procedure is more likely associated with perilymph loss, acoustic trauma, bone dust-related inflammatory reaction followed by osteoneogenesis, or spiral lamina damage than in the case of round window insertion [23]. Conflicting results regarding residual hearing preservation with each approach concede no clear benefit of a particular surgical approach for cochlear implantation [15,25,26]. An ongoing clinical randomized controlled trial (CIPRES) is being conducted with the aim of clarifying this aspect by comparing hearing preservation following cochlear implantation, considering four frequent combinations of surgical approaches (round window and cochleostomy) and electrode array designs (straight and pre-curved) [27].

### 2.3. Robot-Assisted Insertion System

Robotics in neurotology is a relatively recent subject is intended to help with various aspects of cochlear implant surgery, such as drilling a keyhole route to the middle ear for implants, inner ear access, and electrode insertion into the cochlea [28]. Insertion speeds are proportionately correlated with insertion forces, and force peaks appear to be linked to interrupted insertions [29]. Robot-assisted electrode insertion seeks to limit human involuntary tremors and augment accuracy during micromanipulation of the electrode array.

Early experimental studies on cadaveric cochleae revealed that robotic-assisted insertion systems reduced cochlear trauma associated with CI electrode insertions compared to manual insertions [30,31]. Comparative X-ray microscopy (XRM) allowed for direct measurement of the insertion trauma by identifying the cochlear lesions present after the insertion and categorizing them according to a scale of severity first described by Roland and Wright [32].

Following the first reports of cochlear implantations using robot-assisted electrode insertion, the results obtained after a radiological and audiological evaluation showed that this technique is less traumatic [28,33,34]. As was expected, different electrode array (EA) designs delivered different results. Daoudi et al. considered straight and pre-curved EA in their study. Cochlear reconstructions using 3D computed tomography imaging detected that in the case of a straight EA, scalar translocations occurred in 19% of the robot-assisted electrode insertion group and 31% of the manually inserted electrode group. With a pre-curved EA, scalar translocation was present in 50% of the robot-assisted group compared to 38% in the manually inserted electrode group. This comparison offers helpful information to consider when deciding which surgical approach is most advantageous to achieve the desired electrode array design [35].

Torres et al. also described their experience using straight and pre-curved electrodes but did not correlate the electrode type results, instead emphasizing the electrode array’s scalar translocation and functional outcomes. They observed no differences in speech perception in a sound-isolated room between manual electrode array insertion and robot-assisted techniques. However, they did observe that robot-assisted insertion reduced the number of translocated electrodes compared with manual insertion [36] but lacked statistical correlation with an improved speech performance.

As this new technique is promising, studies are still needed to determine safety, utility recommendations, and cost effectiveness. Further developments in robotic technologies and more clinical evidence may convert this surgical approach into a feasible, affordable option. Meanwhile, using intraoperative ECochG during CI surgery can offer the surgeon objective feedback during electrode insertion.

Current surgical principles are outlined in Table 1.

## 3. Pharmacological Support

### 3.1. Corticosteroids

The pharmacological substances with the best results in the preservation of post-implantation hearing are yet to be determined. The same also applies to the dose, delivery, and the timing of administration for currently available pharmacological substances.

As the passing of the electrode through the scala tympani initiates a foreign body inflammatory reaction, pharmacological compounds with anti-inflammatory properties are among the most studied. The most well-known and scrutinized pharmacological compounds for this purpose are still corticosteroids. Their efficacy inspired their use in various hearing preservation protocols in an attempt to decrease any incidental loss of hearing [37]. Although extensively used, the timing of the administration of steroids is not standardized with respect to CI. Human and animal studies have shown that perioperative administration of steroids requires high doses to achieve high intracochlear levels [38].

The authors of one study recommend using intravenous (i.v.) dexamethasone one hour before surgery in order to reduce possible surgery-induced hearing loss [39]; others suggest a direct placement onto the RW niche to reduce post-operative threshold shifts [40,41]. A study conducted by Skarzynska et al. in 2021 on 29 patients assessed two different regimes of steroid therapy. The authors assessed the effect of pre-operative i.v. dexamethasone (DEX) versus i.v. DEX plus oral prednisone versus control in adult patients who underwent cochlear implantation with the Oticon Medical Neuro cochlear implant system (Neuro Zti implant, Neuro 2 sound processor, and Zti EVO atraumatic electrode) [42]. The authors observed significant deterioration of hearing thresholds after CI in all three groups, with no significant improvement at 12 months after surgery, regardless of the medication regime.

Although topical administration of steroids hypothetically implies higher intracochlear concentrations [43], animal studies have demonstrated diminished levels, with the most significant concentrations measured basally [44]. Additionally, more extended periods were found to be necessary to reach peak concentration. The maximum concentration was reached after one hour, and the effect lasted less than 24 h [45].

### 3.2. Brain-Derived Neurotrophic Factor (Neurotrophin)

To obtain the best functionality from a cochlear implant, preservation of the spiral ganglion neurons (SGN) is also of utmost importance, along with the cochlear components, as they are the target of electrical stimulation [46]. Rejali et al. noted that locally applied brain-derived neurotrophic factor (BDNF) did not maintain the viability of the supporting cells, which are associated with improved survival of the neurons. Still, neuronal survival was sustained in the electrode insertion trauma (EIT) and BDNF groups, leading to the supposition of the effectiveness of the BDNF in preserving the function of the spiral ganglion [47]. The same observation was noted by Warnecke et al., in both in vitro and in vivo experiments. The authors coated the electrode with cells secreting BDNF and observed enhanced SGN survival [48].

### 3.3. Glial-Cell-Line-Derived Neurotrophic Factor (Neurotrophin)

Glial-cell-line-derived neurotrophic factor (GDNF) and BDNF attached to the cochlear implant electrode via biodegradable calcium phosphate hollow nanospheres also induced spiral ganglion nerve axons to sprout and grow towards the electrode to form direct contact and close the anatomical gap between them [49].

### 3.4. Neurotrophin-3 (Neurotrophin)

Neurotrophin-3 (NT-3) was studied alongside BDNF or as a standalone substance. Through an experimental animal study using guinea pigs, Pfings et al. delivered intracochlear NT-3 via a viral vector, followed by cochlear implantation. Results were compared to empty viral vector inoculation and cochlear implantation, as well as cochlear implantation without inoculation. Overall results show that NT-3 cochlear inoculation was effective in maintaining a higher number of SGN compared to implanted cochleae with empty virus inoculation and implanted cochleae with no inoculation. Unfortunately, in the subjects with NT-3 inoculation, SGN preservation in the vicinity of the cochlear implant variated from 4% to 49% in animals with no residual hearing and between 4% and 67% in animals with residual hearing. These inconsistencies in SNG density could be an effect of NT-3 or could be derived from electrical stimulation of the implant, which is known to sustain nerve function; therefore, further studies with no electrical stimulation are needed [50].

### 3.5. Insulin-like Growth Factor

Various studies have been conducted on the mechanism of insulin growth factor 1 (IGF-1) and its role as a neurotrophic agent with anti-inflammatory properties capable of inhibiting apoptosis and promoting cell regeneration, making it a potential inner ear protector [51,52,53,54]. Clinical trials demonstrate that IGF-1 can improve hearing after sudden sensorineural hearing loss [55] but is less effective in mitigating ototoxic drug-induced sensorineural hearing loss [56]. When used in combination with a cochlear implant, either as an eluting gelatin hydrogel coating the electrode [57] or placed locally on the round window membrane in the form of soaked porcine skin gelatin [58], IGF-1 demonstrated protection of the auditory brainstem response (ABR) thresholds and showed better histopathological scores compared to a cochlear implant electrode with no coating.

### 3.6. MAPK/JNK Pathway Inhibitor (Antiapoptotic)

Inhibitors of the c-Jun N-terminal kinase-like CEP-1347, SP 600125, and D-JNKI-1 have been investigated as antiapoptotic substances in preclinical and clinical studies since 1900 [59,60,61]. D-JNKI-1(brimapitide/AM-111/XG-102) is a highly selective, non-competitive, long-acting inhibitor of c-Jun N-terminal kinase (JNK); Eshraghi et al. investigated this compound in various preclinical studies [62,63,64,65]. Their results suggest that D-JNKI-1 is a potentially significant protective substance against electrode-induced trauma of the cochlea, both for the acute traumatic phase and the prolonged inflammation phase. Both DPOAE (distortion product otoacoustic emission) and ABR had fewer changes after electrode insertion trauma when associated with D-JNKI-1 [62,64].

D-JNKI-1was investigated in a phase III clinical trial, HEALOS (ClinicalTrials.gov Identifier: NCT02561091), for acute sensorineural hearing loss (ASNHL). The intratympanic AM-111 administration was well tolerated and safe but failed to meet the study’s primary endpoint. Analysis of pure-tone average (PTA) scores revealed significant hearing recovery in the profound idiopathic sudden sensorineural hearing loss (ISSNHL) subpopulation but not in the severe ISSNHL subpopulation [66]. To date, no clinical trials have been conducted that associate this compound with CI, although such studies remain a prospective possibility.

### 3.7. Pioglitazone (Antioxidant)

Pioglitazone is an antidiabetic drug of the thiazolidinediones (TZDs) class used to treat type 2 diabetes mellitus. TZDs act by activating peroxisome proliferator-activated receptors (PPARs), a group of nuclear receptors exerting an anti-inflammatory and antiproliferative effect, as well as regulating the cellular redox balance. When used with gentamicin in in vitro studies conducted by Sekulic-Jablanovic et al., pioglitazone demonstrated a protective cochlear effect against oxidative stress and prevented gentamicin toxicity [67,68]. As PPARs are involved in noise exposure cochlear damage [69], Paciello et al. used pioglitazone in a biocompatible thermogel via transtympanic injection in rats with noise-induced hearing loss [70]. The authors documented a diminished ABR threshold shift and decreased hair cell loss in the middle-basal turn, but no considerable effect was noted in the apical cochlear turn.

In 2018, pioglitazone was included as a 1.2% gel injection compound in a phase III multicentric, double-blind, placebo-controlled clinical trial (ClinicalTrials.gov Identifier: NCT03331627) including adults with sudden sensorineural hearing loss. Participants were randomly allocated to one of three groups: intratympanic pioglitazone gel injection (STR001-IT) followed by 12 weeks of 5mg sustained-release oral pioglitazone tablets (STR001-ER), intratympanic pioglitazone followed by 12 weeks of oral placebo treatment, and intratympanic placebo injections followed by 12 weeks of oral placebo treatment. Results have yet to be published [71]. No preclinical or clinical studies have been conducted to date using this compound concurrently with cochlear implantation; thus, such a combination could serve as a premise for future research.

Other PPAR activators, such as fenofibrate, rosiglitazone, and other antidiabetic drugs, including metformin, are mentioned in the literature as having an otoprotective effect, but none of them have been studied in conjunction with cochlear implants [67,72,73,74,75].

### 3.8. N-Acetyl Cysteine (Antioxidant)

N-acetyl cysteine (N-ACC) is a precursor and the substrate for glutathione synthesis, an endogenous antioxidant. When studied with noise-induced cochlear damage, N-ACC reduced reactive oxygen species (ROS), protected mitochondria, and inhibited inflammation and necrosis [76]. When studied in vitro on cochlear explants, N-ACC showed no toxicity and a protective role against cochlear implant electrode insertion trauma and TNF-α exposure-induced oxidative stress [77,78,79].

An in vivo animal study conducted by Eastwood et al. using a pledget loaded with N-ACC applied on the round window for 30 min before cochleostomy and electrode insertion brought up some detailed information. Whereas local N-ACC exposure led to a non-significant change in threshold shift in the low and mid frequencies, the high frequencies exhibited an increased threshold shift in the first week after surgery, with complete resolution by the fourth week compared to saline. Although there were no signs of active inflammatory reaction, the experimental group did display signs of ossification [80].

The results of a 2021 in vivo study contradicted the favorable effects of N-ACC in vitro. Jaudoin et al. prepared an N-ACC liposomal gel, which was applied transtympanically one week before and during cochlear implantation. After seven days, the hearing thresholds increased in all frequency ranges compared with the group with no N-ACC. When tested on normal hearing guinea pigs, the liposomal gel induced a hearing loss that remained constant until the following two weeks of the study [81]. These observations could restrict its medical use as a local protective agent during cochlear implantation and electrode insertion [82,83].

### 3.9. Taurodeoxycholic Acid (TDCA)

Taurodeoxycholic acid, a bile acid taurine conjugate of deoxycholic acid, was used by Shah et al. in an in vitro study mimicking EIT. By analyzing apoptosis, levels of ROS, nitric oxide, and hair cell (HC) density, the authors observed that TDCA protected against HC loss in a dose-dependent manner [84].

A summary of all the pharmaceutical agents described above is presented in Table 2.

## 4. Drug-Eluting Electrodes

When opting for a cochlear implant as a therapeutic solution for hearing loss, access to the inner ear is created through the round window or a cochleostomy; therefore, the electrode can also support local drug delivery to reduce EIT in order to avoid high systemic drug concentrations with possible adverse effects, blood labyrinthine barrier (BLB) limitation, or the “first-pass” effect [44]. The desired substances can be used to coat the electrode, incorporated into the electrode material, or delivered through an interior channel [44].

Glucocorticosteroids were intensively studied to reduce EIT and DEX-eluting electrodes, resulting in a considerable amount of research data [85,86,87,88,89,90]. In an animal model, Douchement et al. used electrodes embedded in a silicone matrix loaded with various concentrations of DEX and compared them to simple electrodes, evaluating the hearing thresholds at 4–6 weeks after surgery and one year after surgery. At both time points, after cochlear implantation, there was a statistically significant difference in the preservation of residual hearing in favor of the electrode arrays loaded with dexamethasone compared to passive electrodes. The difference was more pronounced at higher frequencies (16,000 Hz) than at lower frequencies [91]. 

Chen et al. used an electrode coated with polycaprolactone (PCL) loaded with 5%, 10%, and 20% DEX to obtain prolonged release. In an artificial perilymph, the electrode released a stable DEX concentration for up to 270 days with no toxicity for the HEI-OC1 cell line. At five weeks after CI, they measured a minor ABR threshold shift in favor of the PCL-DEX-coated electrode when compared to the uncoated electrode. They also observed reduced cochlear fibrosis and better preservation of the SGNs [92].

The first human study with a dexamethasone-eluting cochlear implant electrode elaborated by MED-EL (CIDEXEL) is an ongoing clinical trial that began in June 2020 (ClinicalTrials.gov Identifier: NCT04450290), with no results available yet.

As SGNs seem to be protected by brain-derived neurotrophic factor (BDNF) or neurotrophin 3 (NT3), implanting electrode arrays coated with fibroblasts overexpressing these neurotrophins appears to be a therapeutic alternative for EIT and residual hearing preservation [47,50].

PLGA-coated electrode arrays treated with DEX, aracytine (Ara-C), Ara-C+DEX, and nicotinamide adenine dinucleotide (NAD+) were used recently in an animal study [93]. The ABR threshold of the NAD+ group continuously increased, suggesting that the treatment had no protective effect on residual hearing after CI, whereas the ABR threshold decreased in the DEX, Ara-C, and Ara-C+DEX groups seven days after surgery. The combination of Ara-C+DEX resulted in no cumulative improvement, as its components may target the same signaling pathway.

Hydrogel-coated electrodes filled with recombinant human IGF1, recombinant human growth factor (HGF), a mixture of both were implanted into guinea pig cochleae by Kikkawa et al. [57]. The coated electrodes were associated with decreased ABR thresholds. Simple hydrogel coating exhibited a protective effect compared to the bare electrode. The mixture of IGF1 plus HGF only showed a protective effect for 16 kHz ABR compared to the single compounds, which resulted in a reduction in the ABR threshold over the complete frequency range. The authors also proposed that the hydrogel coating could be used as a natural sealant for the electrode to prevent cochlear perilymph leakage.

## 5. Nanoparticles

As researchers noted that systemic drug delivery into the cochlea was hindered by the limitations mentioned earlier and that local RW application limited permeation into the inner ear, new methods of drug deliveries emerged. One of the main focuses is on loading drugs into/onto nanoparticles (NPs). The use of these nanovectors has been investigated due to their promise for improved drug stability, increasing drug storage capacity, and improving target specificity [94].

BDNF is a macromolecule whose delivery to the inner ear can improve the electrode–neural interface after a cochlear implantation. Nanoporous silica and nanoporous poly-L-glutamic acid (PGA) nanoparticles were studied for this task [95]. Wang et al. described the preparation of a drug transporter based on mesoporous silica (MS) for more effective drug delivery to the inner ear. By applying capillary forces on MS nanoparticles, they were bound into droplets, resulting in a mesoporous silica supraparticle (MS-SP) loaded afterward with BDNF [96]. Using the same loading method as Wang, Wise, et al. used MS-SP for BNDF intracochlear delivery by directly introducing them into the cochlea [97]. Both Wise and Wang detected improved SGN survival than the in ears treated with unloaded supraparticles, with no local toxicity; however, neither of the authors tested ABR thresholds.

Poly-L-glutamic acid can also be used as a building block for BDNF carriers. Nanoporous PGA particles encapsulating BDNF demonstrated a protective effect on the spiral ganglion neurons after intracochlear administration [98]. No studies have compared the efficacy of these two particles or their protective effect in conjunction with CI.

One study was intended to improve the functionality of cochlear implantation using rolipram delivered into the cochlea through a lipidic nanocapsule. An interesting observation of this study was that rolipram alone did not to improve ABR results, but the lipid nanocapsule containing rolipram increased the spiral ganglion survival rate [99].

## 6. Stem Cells

The aim of regenerative medicine applied to hearing loss is to restore hearing without the need for a hearing aid. Currently, regenerative therapies are combined with cochlear implants to improve their functionality. When used as monotherapy, stem cells offer limited benefits and have reduced capacity to restore cochlear function [100].

As repopulation of the cochlea with hair cells with the help of stem cells is technically complex, increasing the population of spiral ganglion neurons appears more feasible in the initial stages. Following this principle of SGN repopulation, stem cells can be associated with CI to achieve better outcomes. BDNF-overexpressing mesenchymal stem cells (MSCs) introduced into a guinea pig cochlea as a CI electrode coated with ultra-highly viscous alginate were more effective in protecting SGNs from degeneration compared to BDNF injected before CI insertion [101].

Roemer et al. described a protocol for elaborating biohybrid cochlear electrodes using autologous mononuclear cells derived from bone marrow (BM-MNC). The authors chose this cellular line for its immunomodulation, neuroprotection, neurorestorative, and neurogenesis properties. Before clinical use, they evaluated cytotoxicity in an in vitro model and studied the insertion forces in a 3D human cochlear model. Three candidates for contralateral cochlear implantation were included in the clinical trial. The authors compared the performance of the biohybrid electrode to that of the contralateral standard non-coated cochlear implant. Whereas none of the patients manifested adverse reactions and the electrode impedances were identical between the ears, speech perception was similar in both ears in just one patient. A second patient’s speech perception in the biohybrid electrode-implanted ear exceeded that in the contralateral ear, whereas speech perception was reduced in the third patient [102]. Although these results are insufficient and inconclusive, they open the door to regenerative therapies associated with CI.

## 7. Gene Therapy

With the advancement of gene sequencing methods, we can now identify a consistent number of genes linked to inherited hearing loss [103]. As hearing aids cannot restore inner ear function, targeted gene regulation can be used as a therapeutic technique. However, this technology is in its infancy, and safety and moral issues are still being debated.

In order to deliver in vivo cochlear gene therapy, a vector is needed. Two main platforms can be differentiated: viral vectors (adenoviruses, adeno-associated viruses, and retroviruses) and non-viral vectors (nanoparticles, exosomes, etc.) [104]. The unique BLB surrounding the cochlea may make it difficult for intravenous or intraperitoneally administered drugs to reach the inner ear, even for precisely targeted vectors [105].

As some of these vectors can be administered transtympanically for an indirect diffusion into the inner ear, a direct access into the cochlea is desirable through the round RWM, the oval window (OW), cochleostomy, or through a semicircular canal.

Gene therapy was combined with a cochlear implant by Pinyon et al. in 2014, using a guinea pig model [106]. They used the cochlear implant electrode array to generate “close-field” electroporation (CFE) to mediate BDNF gene delivery into the cochlea. Cochlear electroporation is known as the transfer of genetic material into the inner ear cells via a current that briefly enhances the permeability of cell membranes. The authors employed a neurotrophin-encoding plasmid DNA vector, which was forced into the cells by a 20 V electrical discharge from the hearing implant, which opened the pores of the cells and allowed plasmids from the surrounding media to be integrated. The use of BDNF gene therapy resulted in the regeneration of neurite processes, which allowed them to grow longer and closer the cochlear implant electrodes, resulting in improved performance as measured by ABR recordings.

Neurotrophin-3 and BDNF have both been proven to be important for SGN growth, survival, and regrowth [50,106,107]. Gene delivery vectors targeting sensory cells within the scala tympani or SGNs could be used to improve future cochlear implants, as SGN preservation is critical for improving the electrode interface of cochlear implants. Improved SNG density results in better neuronal transmission and auditory performance by reducing impedance and current spread [105].

## 8. Conclusions

Simultaneous hearing loss restoration strategies are being developed as we witness rapid technological and biological progress. All of these efforts combined and implemented could greatly benefit patients in the future.

Improved electrode designs, surgical advancements, and microsurgical and robotic instruments will make otologic surgical procedures less traumatic, more accurate, and tailored to each patient’s unique anatomy and needs. Surgical principles are clearly outlined based on current concepts. In the case of robotic surgery utility, guidelines need to be further presented, and cost effectiveness has to be taken into consideration. Locally applied hypothermia is a feasible option due its current use in other surgical areas and due to the fact that is does not present a side-effect profile.

As new drugs with otoprotective properties are studied and new implant materials are developed, cochlear implant drug delivery opens new possibilities for the immediate short-term future. To date, many of the researched substances have not been tested in clinical trials. The only pharmaceutical agent being tested in a clinical trial is MAPK/JNK pathway inhibitor, but the study is not associated with cochlear implantation. Corticosteroids are the only compounds that are frequently used as a potential agent for the preservation of residual hearing, but no clear evidence suggests that one administration route or dose is more favorable than the others.

When improved understanding of nanotechnologies, gene treatments, and stem cells is achieved, construction of a bionic ear could become more compelling. At present, most of these therapies are experimental, as there are still ethical issues (stem cell therapy), as well as toxicity, stability (nanoparticles), and efficiency issues to be addressed.

## Figures and Tables

**Table 1:** Summary of surgical principles favoring the maintenance of residual hearing.

Investigated Principle	Type of Study	Summarized Results	Conclusions
“Soft surgery”	Animal and human studies [2,3,4,5,6,7,8]	Limit drilling due to high SPL and vibration; avoid bone dust and blood entering the inner ear; limit suctioning to avoid perylimph aspiration.	Literature is clear on core surgical principles, which are already used in clinical practice; little evidence on cochlear blood contamination causing SNHL is available.
Substances for facilitating electrode insertion	Animal and human studies [3,9,10,11,12,13,14]	HA is favored to reduce friction and facilitate electrode insertion; other lubricants, such as oxycellulose and glycerine, are not recommended.	Although extensively researched, some data suggest that HA does not offer any benefit with respect to hearing preservation; due to the safety profile, HA can be used for electrode insertion; oxycellulose and glycerine are contraindicated as an electrode lubricant due to foreign body reaction and low impedance.
Partial electrode insertion	Human study [16]	Candidates for electro-acoustic stimulation can benefit from partial electrode insertion.	If hearing further deteriorates, the electrode can be inserted further; insufficient research data are available; the benefit would be the protection of lower frequencies.
Electrode insertion route	Human and animal study [15,23,24,25,27]	The round window approach adheres more to the “soft surgery” principles, but functional outcomes can be similar if cochleostomy is chosen for selected cases.	The round window approach offers no clear benefit in preserving residual hearing. The ongoing multicentric CIPRES study aims to clarify the details.
Electrode type	Human studies [35,36]	Straight and curved electrodes were evaluated, with no clear benefit favoring one electode type over the other.	Electrodes have to be precisely fitted for each individual patient. Electrode design and improvement is a continuously evolving field.
Robot-assisted systems	Human studies [28,31,32,33,34]	Robotic CI limits tremors and damage to the cochlea during electrode insertion (histologic and audiologic data).	Robotic CI results in fewer translocated electrodes but is not correlated with speech perception after implantation. There is a need to determine particular use cases and the cost effectiveness of the approach.
ECochG	Human study [17]	The use of ECochG can ease atraumatic electrode insertion by objective feedback on amplitude and phase response.	ECochG could facilitate the preservation of residual hearing by limiting mechanical trauma during insertion.
Local hypothermia	Animal and human studies [18,19,20,21,22]	Local application of controlled hypothermia has a protective effect.	Only preclinical studies are available, but wider use is being investigated; as it is a non invasive method, it may become an early adition to current principles.

Abbreviations: SPL = sound pressure level; SNHL = sensorineural hearing loss; HA = hyaluronic acid; CI = cochlear implantation; ECochG = electrocochleography; Animal study = in vivo or in vitro; Human study = cadaver temporal bone specimens and clinical cases or trials.

**Table 2:** Summary of pharmacological options for increasing residual hearing following cochlear implantation.

Investigated Substance	Study Type	Summarized Results	Conclusions
Corticosteroids	Animal and human studies [39,40,41,42,43,44,45]	The timing, dose, and administration route are not standardized; higher doses are required for effect; oral and i.v. regimes were studied, but residual hearing deterioration was still noted; topical administration does not yield higher cochlear concentration.	Although they are the most studied substance, there is no compeling evidence that corticosteroids would preserve residual hearing following CI.
Brain-derived neurotrophic factor	Animal and human studies [46,47,48]	BDNF did not sustain the viability of the supporting cells of the SNG but still managed to induce better survival of the neurons than in the control gorups.	No functional data are available; the local use of BDNF needs to be further investigated, but current data may support its furure use.
Glial-cell-line-derived neurotrophic factor	Animal study [49]	Electrodes coated with GDNF and BDNF favor SNG axon growth and contact with the implant electrode.	Only one study shows potential benefits, and no functional data are available; more data are needed in order to draw conclusions.
Neurotrophin-3	Animal study [50]	NT-3 maintained a higher number of SNGs compared to the control group, but results were inconsistent.	More studies are needed to sustain the use of NT-3, both including and exluding electric stimulation.
Insulin-like growth factor	Animal and human studies [55,57,58]	Good results were achieved with SNHL; IGF-1-coated electrodes showed better ABR results when compared to controls.	Limited data are available with promising results; many articles support the neuroprotective effects of IGF-1, but available data need to be transposed to the preservation of residual hearing.
MAPK/JNK pathway inhibitor	Animal and human studies [55,62,63,64,65]	Confirmed as a protective substance for electrode-induced cochlear trauma; ABR showed limited variation after electrode insertion trauma when associated with D-JNKI-1; clinical trials show promising results in profound sudden SNHL.	Insufficient data are available regarding the preservation of residual hearing; data are promising regarding other types of hearing loss; phase III clinical results for profound SNHL suggest MAPK/JNK pathway inhibitors are safe to use.
Pioglitazone	Animal and human studies [67,69,70,71]	A protective effect was achieved in gentamicin-induced hearing loss; a protective effect was observed in noise-induced hearing loss; no studies are available on residual hearing loss.	Promising results were obtained in SNHL, which can be applied in CI.
N-acetyl cysteine	Animal studies [80,81,82,83]	N-acetyl cysteine was protective against noise-induced cochlear damage and found to be safe to use; it was also found to be protective against electrode insertion trauma; in animal models, intracochlear N-ACC use resulted in signs of ossifiacation and hearing threshold increase, as well as hearing loss.	Inconclusive and negative findings may limit its use in N-ACC in CI and otology.
Taurodeoxycholic acid	Animal study [84]	An EIT mimicking in vitro study showed TDCA protection against hair cell loss in a dose-dependant manner.	Only information from an in vitro study is available; more data are needed to sustain the use of TDCA.

Abbreviations: CI = cochlear implant; BDNF = brain-derived neurotrophic factor; SNG = spiral neuron ganglion; NT-3 = neurotrophin; SNHL = sensorineural hearing loss; ABR = auditory brainstem response; IGF-1 = insulin-like growth factor 1; N-ACC = N-acetyl cysteine; EIT = electrode insertion trauma; TDCA = taurodeoxycholic acid.

## Data Availability

Not applicable.
